# Do Family Interventions Improve Outcomes in Early Psychosis? A Systematic Review and Meta-Analysis

**DOI:** 10.3389/fpsyg.2017.00371

**Published:** 2017-03-27

**Authors:** Melanie Claxton, Juliana Onwumere, Miriam Fornells-Ambrojo

**Affiliations:** ^1^Department of Clinical, Educational and Health Psychology, University College LondonLondon, UK; ^2^Department of Psychology, King's College, London, Institute of Psychiatry, Psychology and NeuroscienceLondon, UK

**Keywords:** family intervention, early psychosis, schizophrenia, relapse, expressed emotion, caregiver burden, meta-analysis

## Abstract

Family interventions for psychosis (FIp) are effective in reducing service user relapse and carer distress in people with schizophrenia-spectrum conditions. Several treatment and best practice guidelines recommend FIp for all people with schizophrenia. However, outcome findings in relation to early psychosis groups have been inconsistent. The current paper reports a systematic review and meta-analyses of articles that evaluated FIp in early psychosis with a clearly defined comparison group. A combination of electronic database searches (using PsychINFO, Medline, and CENTRAL), citation searches and hand searches of key journals and reviews was conducted. Peer-reviewed articles published in English from database inception to June 2016 were included. Methodological quality was assessed using the Effective Public Health Practice Project Quality Assessment Tool (EPHPP). Seventeen papers from 14 studies met inclusion criteria for review, the overall quality of which was moderate. Meta-analytic synthesis showed that FIp improved service user functioning and reduced the likelihood of relapse by the end of treatment. Psychotic symptoms were significantly reduced in the FIp group at follow up, but this was not evident at end of treatment. In terms of FIp target mechanisms, carers receiving FIp were more likely to shift from high to low expressed emotion and less likely to report patient focused criticism or engage in conflict communication than carers randomized to standard care. Carer burden and well-being were improved by the end of treatment but gains were not sustained at follow up. FIp had no impact on carer emotional over-involvement. The findings indicate that FIp is an effective intervention for early psychosis service users and their relatives. However, further research is required to establish which key therapeutic components of FIp are most effective for whom, in addition to understanding the mechanisms by which FIp might affect positive change.

## Introduction

The first episode of psychosis usually occurs in late adolescence to early adulthood (Mueser and McGurk, 2004). The few years following the emergence of symptoms are considered to be a “critical period”; involving the greatest clinical deterioration (Lieberman et al., 2001), determining the future course and prognosis of the illness and offering a window for ensuring optimal support and treatment (Birchwood et al., 1997). Delays in accessing treatment after the onset of psychosis have a median of 8–11 months (Norman et al., 2001; Morgan et al., 2006) with longer duration being associated with poorer outcome (Perkins et al., 2005). Moreover, service users with first episode of psychosis report high levels of trauma (Duhig et al., 2015), commonly present with self-harm (Harvey et al., 2008), social and vocational difficulties (Sündermann et al., 2013; Fornells-Ambrojo et al., 2014).

Diagnostic uncertainty can often follow a first episode and long-term outcomes are unclear (Addington et al., 2006). An increased number of episodes (or relapses) during the early stages of psychosis is associated with poorer clinical (Birchwood et al., 1998; Emsley et al., 2008) and recovery outcomes (Shrivastava et al., 2010). There is a continued need for early identification and effective treatment options to support those who might be at risk of developing psychosis, as well as those in the early stages of the illness, in order to ensure optimum prognosis (McGlashan et al., 2007).

Family support is particularly relevant for the early psychosis group as the onset of illness often occurs at a time when many young people are still living at home (Garety and Rigg, 2001; Fisher et al., 2008; Jansen et al., 2015). Evidence confirms that family support can be linked to significantly fewer rates of relapse and rehospitalisation (Norman et al., 2005), improved mortality (Revier et al., 2015) and treatment engagement (Stowkowy et al., 2012). Family members are usually the first to notice changes and identify indicators of relapse and crisis (Addington and Burnett, 2004; Jackson and McGorry, 2009). Crucially, the manner in which family members respond to the condition has considerable influence on illness course. High levels of critical comments, hostility and/or emotional over-involvement in family members (commonly known as high expressed emotion), are associated with poorer patient outcomes including more frequent relapse, and hospital admissions in people with longstanding psychosis (Bebbington and Kuipers, 1994; Butzlaff and Hooley, 1998; Cechnicki et al., 2013) and relatives reporting higher carer, burden of care, and less adaptive coping strategies (Raune et al., 2004; Kuipers et al., 2006).

Family interventions for psychosis (FIp) are evidence based talking therapies that are known to significantly reduce relapse and hospital readmission rates for people with psychosis and improve levels of medication adherence and social functioning (Pitschel-Waltz et al., 2004; Pharoah et al., 2006, 2010). The interventions have also been linked to significant improvements in carer outcomes that include reduced care burden and increased readiness to provide care (Berglund et al., 2003; Lobban et al., 2013). Family interventions vary in their modes of delivery (multifamily vs. individual family, sessions held within family home or clinic, service user involved or not); however, they share key components (Barrowclough and Tarrier, 1992; Kuipers et al., 2002; Addington and Burnett, 2004). These include information sharing (psycho-education), problem-solving, emotional processing, stress management and communication. The components are designed to facilitate an improved understanding about psychosis and the emotional impact of the illness on family relationships, promote more adaptive coping and minimize risk of relapse (Onwumere et al., 2011). FIp is cost-effective (Mihalopoulos et al., 2004) and included in treatment and best practice guidelines (Gaebel et al., 2005; International Early Psychosis Association Writing Group, 2005; Kreyenbuhl et al., 2010; IRIS, 2012; NICE, 2014).

In comparison to longer-term illness groups, there have been fewer FIp studies in first episode psychosis (FEP) groups and the evidence base on its efficacy during this illness phase is somewhat more limited and equivocal. In a previous systematic review, Bird et al. (2010) examined a small number of randomized controlled trials (*n* = 3) within specialist early intervention for psychosis services and found that service users whose families received FIp were less likely to relapse or be admitted to hospital at the end of treatment, compared to those receiving standard care. All other reviews examining the efficacy of FIp in early psychosis report mixed findings (Penn et al., 2005; Askey et al., 2007; Sadath et al., 2015). However, there have been a number of limitations to previous reviews, including not using a systematic search strategy (Askey et al., 2007), only examining RCTs taking place within specialist early intervention for psychosis services (Bird et al., 2010), including mixed-length illness samples (Pharoah et al., 2010) and multi-element interventions (Penn et al., 2005) or limiting the review period to the last two decades (Sadath et al., 2015). Further, with exception of Bird et al. (2010), no previous reviews have employed a meta-analytic approach to provide quantitative synthesis of the evidence.

The current review aimed to systematically review the available literature on FIp and examine whether FIp improves outcomes for service users and carers within an early psychosis population using meta-analytic synthesis. In particular, we sought to answer the following questions:

1. Does FIp reduce the risk of relapse and improve symptoms and functioning in service users with early psychosis?
2. Does FIp reduce high expressed emotion (criticism, hostility and emotional over-involvement)?
3. Does FIp in early psychosis reduce burden of care and improve carer well-being?

## Methods

### Search strategy

The Preferred Reporting Items for Systematic Reviews and Meta-Analyses (PRISMA; Moher et al., 2009) guidelines were followed in conducting this systematic review and meta-analysis. Studies were identified through a combination of computerized database searches, citation searches and manual searches of bibliographies.

A systematic search of the literature for relevant articles published from database inception until June 16th 2016 was performed using the databases PsychINFO (using Ovid), PubMed, and CENTRAL (Cochrane Central Register of Controlled Trials). Results were limited to English language and peer-reviewed journal articles. Preliminary searches using keywords within the categories of “family intervention” and “psychosis or schizophrenia” indicated that these two categories alone were over-inclusive. Restricting papers to those that also included keywords related to “at risk” or “early psychosis” did not change the number of relevant papers retrieved. A list of keywords and MeSH (Medical Subject Headings) terms was generated to identify studies that included family-based interventions for those considered at risk of developing psychosis and those who had experienced recent-onset psychosis. A comprehensive list of search terms was used to capture all variations within each of three categories: (i) psychos^*^ / psychotic^*^ / schizophren^*^, schizoaffective (ii) famil^*^ intervention / famil^*^ therap^*^ / famil^*^ work / psycho education / group intervention / group work / group therap^*^ (iii) early / at risk / high risk / first episode / prodrom^*^ / first onset / critical period. The search returned only papers that contained at least one term from each category (three separate searches were completed and then combined by using the Boolean operator “AND”).

### Eligibility criteria

The criteria for including studies within the review were as follows: (1) studies evaluating a clinician-led family intervention (including family work, psycho-education and family therapy) of any duration; (2) service user population defined as either “at risk” (using validated assessment methods e.g., those with a family history of psychosis or displaying prodromal symptoms) or with a diagnosis of early psychosis (service users described as “first episode,” “early psychosis,” “first admission,” or those service users within the first 5 years of diagnosis); (3) quantitative studies with a clearly defined control or comparison group (for example RCTs or Clinical-Controlled trials) and (4) studies published in English and in peer-reviewed journals (abstracts, reviews, case reports, thesis dissertations, and case studies were discounted).

Studies with no comparison group were excluded. In addition, studies where family interventions were offered as part of an integrated treatment, but where the methodology did not identify, define and report outcomes in relation to a family intervention component were not included. For example, studies that described family interventions as part of a comprehensive early intervention programme, but did not report which service users or carers had received FIp, or only evaluated the entire multi-component programme, were excluded. Papers that reported primary or secondary outcomes related to: (a) service user relapse, symptoms and functioning; (b) carer expressed emotion/family environment, burden and well-being were included. Authors were contacted when studies did not detail sufficient statistical information for the calculation of effects comparable to other studies. When this information was not available and not provided by authors, studies were excluded.

### Assessment of methodological quality

The methodological rigor of each study was assessed using the Effective Public Health Practice Project Quality Assessment Tool (EPHPP, 1998). This tool assesses the quality of quantitative studies, employing different study designs, and across six key domains: selection bias, study design, confounding variables, blinding, data collection methods, and withdrawals and dropouts. Domains are rated as strong, moderate or weak, based on information reported in the paper. A global rating was then calculated and each paper was rated as strong (no weak ratings), moderate (one weak rating), or weak (two or more weak ratings). It has good content and construct validity (Thomas et al., 2004; Jackson and Waters, 2005). Fifty percent of papers were co-rated (by at least two authors; MC, JO, and MFA) with discrepancies in scoring discussed until an agreement was reached.

### Synthesis and data extraction

Following the quality assessment, a synthesis of studies was carried out focusing on participant characteristics, study design, measures and intervention details. For the meta-analyses, data were extracted, where available, for the following service user outcomes: mean symptoms of psychosis; mean level of functioning; number of service users with at least one relapse (using the same criteria as Bird et al. (2010) with relapse defined as a hospital admission or relapse as assessed by significant symptom deterioration indicating an episode of illness); and length of hospital admission. Carer outcomes extracted included: number of carers changing from high to low expressed emotion (EE); mean level of criticism and over-involvement as part of EE assessment; levels of conflict in communication; carer burden and well-being. Outcome data was extracted at both end of treatment and follow up when available.

### Statistical analyses

Meta-analyses were carried out in Review Manager (Version 5.3). Bias-corrected standardized mean difference (SMD) was calculated on continuous outcome measures for every intervention-control group comparison using pooled standard deviation as the standardizer (Hedges and Olkin, 1985). We calculated relative risk (RR) for binary outcomes. Effect sizes were calculated based on post-treatment data and follow-up data in order to estimate the long-term effects of treatment. Homogeneity of effect size was not assumed because the studies differ in various ways (for example intervention content, length and number of sessions and whether FIp was delivered in a group or individual family format). Hence, a random-effects model was fitted to the data to allow for variation in the true effect sizes (δ*i*). Heterogeneity were calculated using χ2 tests and the I^2^ statistic was reported. When *I*^2^ = 0, 25, 50, or 75%, then no, low, moderate and high heterogeneity must be assumed (Higgins et al., 2003). Sensitivity analyses using one-study-removed method were also carried out when heterogeneity was found to be high to examine whether any specific study had an increased influence on the pooled treatment effect (Ryan, 2013). In such cases, clinical and methodological heterogeneity were reviewed in relation to the remaining studies to evaluate if it would be justified to exclude them from the particular meta-analysis. Funnel plots were not produced to examine publication bias given that the larger number of studies in the included meta-analysis was *k* = 7, below the recommended minimum of 10 (Sterne et al., 2011).

## Results

### Study selection

**Figure 1:**
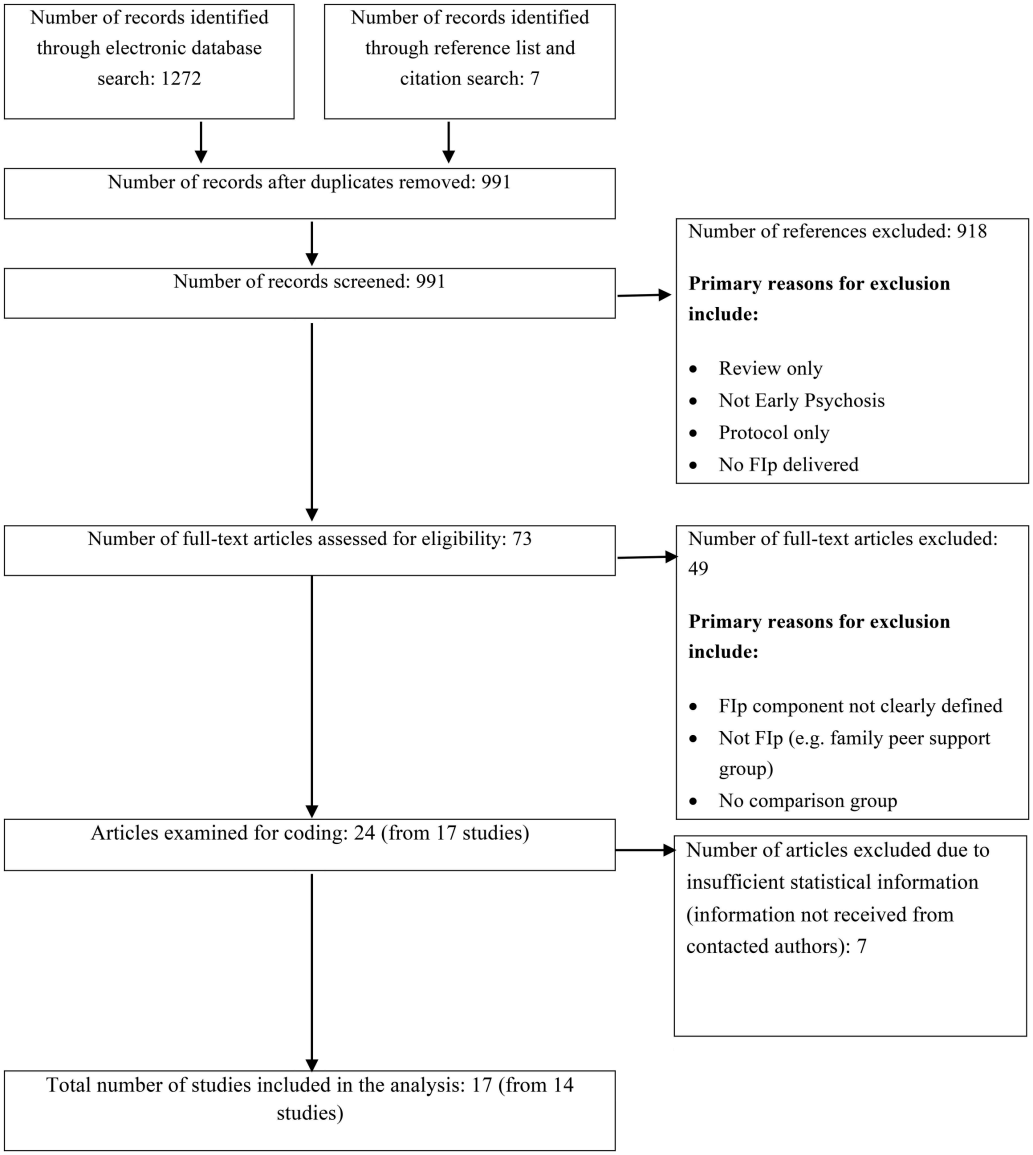
**Study selection and primary reasons for reference exclusion**.

The study selection process is outlined in Figure 1. After removing duplications, the electronic and hand search generated 991 papers, which were screened by title and abstract, after which 918 were excluded. The full-text articles of the 73 remaining papers were read in full and considered against the inclusion and exclusion criteria. Each paper was reviewed by at least two members of the research team (MC, MFA, or JO). Disagreements were resolved via discussion. A further 49 papers were excluded after failing to meet all the eligibility criteria. Primary reasons for exclusion included: (1) the family intervention not being clearly defined in the method or analysis (e.g., reported as part of an integrated, multi-element service, meaning it was not possible to separate FI component in the analysis); (2) no comparison group; and (3) participants not meeting the “early psychosis” criteria. A further 8 papers (from 5 different trials) met the inclusion criteria but were later excluded due to insufficient statistical information to complete the meta-analysis (all authors were contacted to obtain further information but did not respond or were not able to provide the required information). This left a total of 17 papers (from 14 trials) to be included for this review.

### Quality assessment of included studies

Overall, the quality of the studies, as rated by the EPHPP, was moderate to strong. All 17 papers were rated and 8 (47%) were classified as strong, 7 (41%) as moderate and 2 as weak (2%; see Table 1). The design of studies was of high quality, with 88% (*n* = 15) of papers rated as strong in this area. Data collection methods and confounds were also relatively strong domains, with 82% (*n* = 14) and 77% (*n* = 13) of papers respectively rated as strong. Studies used reliable and valid data collection methods and generally reported and controlled for confounds and dropouts (i.e., by using methods such as intent-to-treat analyses). There was, however, some participant selection bias; whilst most studies were representative of the target population, several studies reported <80% participation in the trial following the initial invitation to participate in research, leading to most papers rated as moderate in this area. One paper (Leavey et al., 2004) was rated as weak in this domain due to <60% of individuals agreeing to participate in the study. In the withdrawals/dropouts domain, only 64% of papers were rated as strong due to papers not always reporting numbers or reasons for drop-outs and/or reporting high drop-out rates. Attempts were made to blind the assessing researcher in most studies, but again, this was not always possible, particularly those with longer follow-ups as papers reported service users unintentionally revealed which group they were in. Gleeson et al. (2010) was one of two studies to receive an overall weak rating, which was due to the high dropout rate at the final point of follow up (whilst 75% of participants completed treatment, only 33% of participants completed the 30-month outcome assessment follow up). In addition, Gleeson et al. (2010) reported, but did not control for two confounders (i.e., the FIp group were significantly more likely to be employed and residing with the service user than controls). Rund et al. (1994) received a weak rating due to lack of blinding and unreliable data collection methods (FIp was compared to an historical cohort and different assessment measures were used for treatment and comparison group).

**Table 1:** **Quality assessment of reviewed studies (using the EPHPP) ***N*** = 17**.

**Study (primary author)**	**Selection bias**	**Study design**	**Confounders**	**Blinding**	**Data collection methods**	**Withdrawals and drop-outs**	**Global rating**
Calvo et al., 2014	M	S	S	M	S	S	Strong
Calvo et al., 2015	M	S	S	M	S	S	Strong
Chien et al., 2016a	S	S	S	M	S	S	Strong
Chien et al., 2016b	S	S	S	M	S	S	Strong
Goldstein et al., 1978	S	S	S	M	S	S	Strong
Linszen et al., 1996	S	S	S	M	S	S	Strong
McCann et al., 2013	M	S	S	M	S	S	Strong
Miklowitz et al., 2014	M	S	S	M	S	M	Strong
Browning et al., 2013	S	S	S	W	S	S	Moderate
Cozolino et al., 1988	S	S	S	W	S	S	Moderate
De Giacomo et al., 1997	M	S	W	M	S	M	Moderate
Leavey et al., 2004	W	S	S	M	S	M	Moderate
O'Brien et al., 2014	M	S	S	M	W	M	Moderate
Rossberg et al., 2010	W	M	M	M	M	M	Moderate
Zhang et al., 1994	M	S	W	M	S	S	Moderate
Gleeson et al., 2010	M	S	W	M	S	W	Weak
Rund et al., 1994	M	M	S	W	W	S	Weak

*S, strong; M, moderate; W, weak. Global Rating is calculated using information across all six domains: strong (no weak ratings), moderate (one weak rating), or weak (two or more weak ratings)*.

The EPHPP offers additional scales to assess aspects such as treatment completion rates and intervention fidelity (although this is not including in the overall rating). Of the 16 studies that recorded this information, treatment completion was found to be generally good: 13 studies reported that more than 60% of relatives completed the intended intervention. Treatment fidelity was good, with 15 studies reporting high consistency across the interventions (the remainder of studies did not report whether consistency was monitored or not).

### Study characteristics

This meta-analytic review encompassed data from 17 articles, reporting findings from 14 distinct studies (study characteristics are detailed in Table 2). Fourteen distinct studies will be referred to when summarizing the characteristics, for purposes of clarity. Seven studies were conducted in Europe, three in North America, two in Australia, one in Hong Kong and one in Mainland China. Eleven studies employed randomized controlled designs, one a controlled clinical trial (Browning et al., 2013) and two used an uncontrolled design (e.g., a cohort analytic design; Rossberg et al., 2010 and Rund et al., 1994).

**Table 2:** **Characteristic of Included Trials (***N*** = 14)**.

**Primary author, publication year and country of origin**	N^*^	**Design**	**Patient characteristics**	**FIp description: delivered to individual or groups, key components, manual, *n* analyzed at assessment point(s)**	**Duration of FIp**	**Comparison Group (s)**	**Outcome measures extracted for current meta-analyses: (1)Service user symptoms; (2) Service user functioning; (3)Relapse/ hospitalization/transition; (4) Length of hospitalization; (5) Career from high to low EE; (6) EE: criticism; (7) EE: emotional over-involvement; (8) Conflict in communication; (9) Caregiver burden; (10)Career wellbeing**	**Treatment completion**	**Follow up**
**AT RISK / PRODROMAL PSYCHOSIS**									
O'Brien et al. (2014) and Miklowitz et al. (2014) USA	129	RCT (from larger trial)	Patients (mean age 16.9 years) at risk of psychosis	Single family: Psychoeducation, Communication skills, Problem-solving, Stress management. Adapted Family-Focused Therapy for intervals at clinically high risk for psychosis) Caregiver *n* = 38, *Patient n* = 47	18 × 1 h sessions over 6 m	Enhanced Standard Care (including × 3 psychoeducation sessions over 1month) Caregiver *n* = 28, *Patient =* 55	(1) SOPS (8) Assessment of family communication (clinician-rated based on Bellack et al., 1996)	56%^**^	None
**EARLY PSYCHOSIS**									
Browning et al. (2013) UK	30	CCT	Inpatients, under 18 years, (mean age 16.9 years) psychotic symptoms on admission	Single family: Psychoeducation, Communication skills Based on manuals (Glick et al., 1993; Kuipers et al., 2002) *n* = 9	5 × 1 h sessions over 4–10 weeks	(i) CBT (*n* = 10) (ii) Standard Care (*n* = 9)	(1) BRPS, (2) CGAS	100%	None
**Primary author, publication year and country of origin**	N^*^	**Design**	**Patient characteristics**	**FIp description: delivered to individual or groups, key components, manual**, ***n*** **analyzed at assessment point(s)**	**Duration of FIp**	**Comparison Group (s)**	**Outcome measures extracted for current meta-analyses**	**Treatment completion**	**Follow up**
Calvo et al. (2014, 2015) Spain	55	RCT	Adolescents 14–18 years (mean age 16.5 years) with very early onset psychosis. Max previous hosp admissions = 3	Single family and Group: Psychoeducation, Problem-solving based on manual McFarlane et al. (1995) T1*: n* = 27, T2: *n* = 25	3 × 50-min individual sessions, then 12 × 90-min group sessions, bi-monthly for 6 months	Non-structured group intervention plus Enhanced standard care T1: *n* = 28, T2: *n* = 26	(1) PANSS, (2) CGAS, (8) FES	63.6%	24 month
Chien et al. (2016a,b) Hong Kong	116	RCT	First episode psychosis, less than 6 months onset, mean age 25 years	Single family: Guided self-help, problem-solving based bibliotherapy based on translated manual (McCann et al., 2013) *n* = 56	5 modules completed over 5 m (plus 2 × 2 h engagement, 3 × 1.5 h review sessions and weekly phone calls)	Standard care *n* = 56	(1) PANSS, (4) length of hospital admission, (3) number of patients re-hospitalized, (9) ECI	98.2%	6 month
Cozolino et al. (1988) USA	29	RCT (stratified for High/Low EE)	Patients admitted with psychosis, less than two years onset	Group: Psychoeducation (based on education syllabus. West et al. (1985)) *n* = 14	1 × 3 h session	Standard care *n* = 15	(6) PRS, (8) FCS	100%	None
De Giacomo et al. (1997) Italy	38	RCT	Schizophrenia; duration of less than 3 years	Single family: Systemic Family Therapy *n* = 19	10 sessions, weekly plus 2x follow-up sessions at 3 and 6 months). Final testing at the end of 12 months	Standard Care including pharmacological treatment (average 8.5 monthly sessions with psychiatrist over 1 years) *n* = 19	(5) FMSS	89%	None
Gleeson et al. (2010) Australia	63	RCT (From a larger trial)	First episode psychosis, less than 6 months of treatment and remission of positive symptoms	Single family: Psychoeducation, Communication skills, Problem-solving, Relapse prevention. Based on manuals (Falloon I. R., 1988 and Mueser and Glynn (1999)) T1: *n* = 23, T2: *n* = 10	Minimum of 18 months FIp	Enhanced Standard Care T1: *n* = 25, T2: *n* = 11	(6) FQ, (7) FQ, (9) ECI, (10) GHQ-28	71%	30 month
Goldstein et al. (1978) USA	104	RCT	Early psychosis; all first (69%) and recent second admissions (within a year). Mean age 23.6 years	Single family: Psychoeducation, Relapse prevention *n* = 47 (high drug/low drug)	6 sessions, weekly	Standard Care and randomly assigned to low drug/high drug *n* = 49 (high drug/low drug)	(3) Number of patients re-hospitalized	92%	None
Leavey et al. (2004) UK	106	RCT	First episode of psychosis, less than 6 months onset	Single family: Psychoeducation, Problem-solving, Coping skills (incorporating ideas from manual: Falloon (1984)) *n* = 57	7 × 1 h sessions (at home or convenient place)	Standard care *n* = 49	(3) Number of patients re-hospitalized	42% (full completion), 57.9 % (partial completion)	9 month
Linszen et al. (1996) The Netherlands	76	RCT	Recent onset Schizophrenia; 15–26 years (mean age 20.7 years)	Single family: Psychoeducation, Communication skills, Problem-solving *n* = 37	18 sessions over 12 months (delivered as flexibly as possible)	Enhanced Standard care *n* = 39	(3) Number of patients re-hospitalized	100%	12 month, 5 year
McCann et al. (2013) Australia	124	RCT	First episode psychosis (duration of 2–3 years treatment) 15–25 years	Single family: Problem-solving Bibliotherapy (based on manual written by authors) *n* = 56	5 × Bibliotherapy modules, weekly	Enhanced Standard Care *n* = 58	(6) FQ, (7) FQ, (9), (9)ECI, (10) K-10	100%	4 month
Rossberg et al. (2010) Norway	301	Cohort analytic	First episode psychosis, actively psychotic and no previous treatment (15–65 years)	Group: Psychoeducation, Communication skills, Problem-solving *n* = 147	90 min sessions Bi-monthly over 2 years.	Not offered (e.g., no family) or refused FIp. Enhanced standard Care *n* = 153	(3) Number of patients re-hospitalized	55%^**^	5 year
Rund et al. (1994) Norway	24	Cohort analytic	Adolescents inpatients (13–18 years) with early onset psychosis	Single family and Group: Psychoeducation, problem-solving plus a “low EE” environment on the inpatient unit *n* = 12	Parent seminars (whole day 2–3 per year), problem solving sessions, over 2 years	Standard care from historic cohort: Patients treated at the same hospital but at an earlier point in time (from 1980 to 1987) *n* = 12	(2) GAS, (4) length of readmission, (5) CFI and Clinical Rating	100%	None
Zhang et al. (1994) China	83	RCT	First admission patients with schizophrenia, mean illness duration 2.8 years, males only, mean age 24 years	Group: Psychoeducation and supportive counseling *n* = 39	Minimum × 1 session once every 3 months for 18 months	Standard care *n* = 39	(1) BRPS, (2) GAS	100%	None

* Number of participants randomized or enrolled in trial;

** *Patients who completed at least 50% of the intended intervention; BPRS, Brief Psychosis Rating Scale; CCT, Controlled Clinical Trial, (C) GAS, (Children's) General Assessment Scales; CFI, Camberwell Family Interview, ECI, Experience of Caregiving Questionnaire; FES, Family Environment Scale; FCS, Family Conflict Scale; FMSS, Five Minute Speech Sample; FQ, Family Questionnaire, GHQ-28, General Health Questionnaires; K-10, Kessler Psychological Distress Scale; PANNS, Positive and Negative symptoms Scales; PRS, Patient Rejection Scale; SOPS, Scale of Psychosis-Risk Symptoms*.

#### Participant characteristics

Carers of 1,278 service users were enrolled in the 14 included trials (577 in FIp and 635 in comparison group), with a mean sample size of 91.29 (*SD* = 70.65). One trial (O'Brien et al., 2014 and Miklowitz et al., 2014) examined those at risk of developing psychosis, whilst the remaining 13 examined those with early or first episode psychosis. Where details were recorded, service users were aged between 12 and 40 years old, and three studies exclusively examined service users with “very early-onset” psychosis (those with onset under-18 years old; Rund et al., 1994; Browning et al., 2013; Calvo et al., 2014). Limited information was provided about the identified carers. From the studies that did note this information, they were predominantly parents (81%; four studies recorded this data), with mean ages reported to be between 45 and 47.5 years (three studies recorded this) and generally lived with the person they cared for (80.7%; across four studies).

#### Family intervention

The interventions comprised a mixture of single family work (*n* = 9), group family work (*n* = 3) or a mixture of both (*n* = 2). Some interventions were delivered to carers only (Cozolino et al., 1988; McCann et al., 2013; Calvo et al., 2014; Chien et al., 2016a,b), whilst others invited service users to join all or part of the intervention (Goldstein et al., 1978; Rund et al., 1994; Zhang et al., 1994; Linszen et al., 1996; De Giacomo et al., 1997; Rossberg et al., 2010; Miklowitz et al., 2014; O'Brien et al., 2014). Three studies did not mention whether service users attended the sessions or not (Leavey et al., 2004; Gleeson et al., 2010; Browning et al., 2013).

The content of the interventions differed across trials; seven studies were based on published manualized interventions (i.e., Falloon, 1984; Glick et al., 1993; McFarlane et al., 1995; Kuipers et al., 2002; McCann et al., 2013), whilst the remaining referenced study-specific protocols. Despite the differences, there were shared commonalities: the majority of interventions included psycho-education as a chief component (*n* = 11), and many of these incorporated communication and problem-solving skills training. One study (De Giacomo et al., 1997) used a systemic family therapy intervention, which specifically excluded any psychoeducational component.

In addition to the differing content, the “dose” of intervention also varied between studies. Eleven trials examined a structured family intervention with a specified number of sessions. Of these, Cozolino et al. (1988) was the shortest, comprising a one-off, 3-h psychoeducational workshop. For the remaining 10 of these studies, the total number of sessions ranged from 5 to 18 (mean = 11 sessions) and individual session duration ranged from 60 to 180 min, spanning between 4 weeks and 24 months. Three studies offered less structured session formats, offering flexible sessions over 18–24 months (Rund et al., 1994; Zhang et al., 1994; Gleeson et al., 2010). Six studies compared FIp to enhanced standard care (for example specialist early intervention for psychosis services), seven studies compared FIp to a form of standard care (for example general adult mental health outpatient services) one study compared FIp to standard care plus a non-structured group. Treatment completion rates were generally high (mean = 83.3%).

### Meta-analyses

Table 3 provides a summary of the results of the meta-analyses for the ten selected outcome variables. For details on which study contributed to each of the outcomes please see Table 2 and Supplementary Materials.

**Table 3:** **Analysis of Family Intervention for psychosis (FIp) compared to standard care (random-effects model)**.

	**Outcome**	**Time of data collection**	**Trials k**	**Participants, *n* FIp/control**	**Estimate**	**Summary of estimate [95% CI]**	**Z, *p***	**Favors FIp/control**	**Heterogeneity**	
									**Q test**	**I^2^ (%)**
Service user	(1) Symptoms (BPRS; PANSS, SOPS)	End of treatment	4	129/130	SMD	−0.26 [− 0.61, 0.09]	1.5, *p <* 0.15	–	*Q* = 5.4, *p* = 0.14	45
		Up to 2 years follow-up	2	81/82	SMD	−0.85 [−1.05, −0.20]	2.6, *p =* 0.01^*^	Favors FIp	*Q* = 3.6, *p* = 0.06	72
	(2) Functioning (C/GAS)	End of treatment	4	87/88	SMD	0.74 [0.13, 1.36]	2.4, *p =* 0.02^*^	Favors FIp	*Q* = 10.0, *p* = 0.02	70
		Up to 2 years follow-up	1	25/24	SMD	0.22 [−0.34, 0.79]	0.8, *p =* 0.43	–	n/a	n/a
	(3) Relapse (num. of people hospitalized/relapse in symptoms/transition to psychosis)	End of treatment	7	303/291	RR^1^	0.58 [0.34, 1.00]	0.3, *p =* 0.05^*^	Favors FIp	*Q* = 12.13, *p* = 0.06	51
		Up to 5 years follow-up	3	242/232	RR	0.98 [0.32, 2.99]	0.0, *p =* 0.98	–	*Q* = 9.4, *p* = 0.009	79
	(4)Length of hospitalization throughout treatment /follow-up	End of treatment	3	81/80	SMD	−0.58 [−1.43, 0.27]	1.3, *p =* 0.18	–	*Q* = 6.1, *p* = 0.01	84
		Up to 2 years follow-up	2	33/43	SMD	−0.12 [−0.58, 0.35]	0.5, *p* = 0. 62	–	*Q* = 0.0, *p* = 0.99	0
Carer	(5) *n* of carers changed from high to low EE (CFI, FMSS)	End of treatment	2	27/22	OR^1^	16.76 [1.96, 143.44]	2.6, *p =* 0.01^*^	Favors FIp	*Q* = 0.42, *p* = 0.52	0
	(6) EE: criticism (FQ, PRS)	End of treatment	3	94/97	SMD	−0.84 [−1.15, −0.53]	5.3, *p <* 0.001^*^	Favors FIp	*Q* = 31.1, *p* < 0.001	94
		Up to 2.5 years follow-up	1	10/11	SMD	−0.96 [−1.87, −0.05]	2.1, *p =* 0.04^*^	Favors FIp	n/a	n/a
	(7) EE: emotional over involvement (FQ)	End of treatment	2	79/83	SMD	−0.08 [−2.14,1.97]	0.1, *p =* 0.94	–	*Q* = 33.5, *p* < 0.001	97
		Up to 2.5 years follow-up	2	66/69	SMD	−0.45 [−1.94, 1.04]	0.6, *p =* 0.56	-	*Q* = 8.6, *p* = 0.004	88
	(8) Communication conflict (FCS, FES, and clinician coding)	End of treatment	3	80/70	SMD	−0.44 [−0.77, −0.12]	2.7, *p =* 0.008^*^	Favors FIp	*Q* = 1.4, *p* = 0.51	0
	(9) Caregiver burden (ECI)	End of treatment	3	135/139	SMD	−0.72 [−0.97, −0.47]	5.7, *p <* 0.001^*^	Favors FIp	*Q* = 17.4, *p* < 0.001	88
		Up to 2.5 years follow-up	3	122/125	SMD	−0.31[−1.53, 0.91]	0.5, *p =* 0.62	–	*Q* = 36.6, *p* < 0.001	95

*BPRS, Brief Psychosis Rating Scale; CCT, Controlled Clinical Trial; (C) GAS, (Children's) General Assessment Scales; CFI, Camberwell Family Interview; ECI, Experience of Caregiving Questionnaire; FES, Family Environment Scale; FCS, Family Conflict Scale; FMSS, Five Minute Speech Sample; FQ, Family Questionnaire; GHQ-28, General Health Questionnaires; K-10, Kessler Psychological Distress Scale; PANNS, Positive and Negative symptoms Scales; PRS, Patient Rejection Scale, SOPS = Scale of Psychosis-Risk Symptoms*.

#### Service user outcomes

Family intervention for psychosis did not achieve *symptom reduction* by the end of treatment when compared to control groups, as measured by the Brief Psychiatric Rating Scale (BPRS, Overall and Gorham, 1962), the Positive and Negative symptoms Scales for Schizophrenia (PANNS; Kay et al., 1987), and the Scale of Psychosis-Risk Symptoms (SOPS; Miller et al., 2003). However, the estimated effect size for symptom improvement for up to 2 years follow-up was large (*k* = 2, *d* = −0.85, 95 % CI [−1.05, −0.20]. Conversely, *general functioning*, measured across studies using the Children's/Global Assessment Scales (CGAS, Shaffer et al., 1983; GAS, Endicott et al., 1976), significantly improved for service users in the family intervention group (*k* = 4, *d* = 0.74, 95 % CI [0.13, 1.36]) but such gains were not maintained by follow up. Two of the studies reporting improvements in general functioning were RCTs, had active comparison groups and were rated as moderate to strong on the EPHPP (Browning et al., 2013 and Calvo et al., 2014) whilst one study (Rund et al., 1994) used an historic cohort as a comparison and was rated as weak on the EPHPP.

The *relapse outcome* included: five studies reporting the number of service users who were hospitalized at least once (Goldstein et al., 1978; Rund et al., 1994; Zhang et al., 1994; Leavey et al., 2004; Chien et al., 2016a), one study assessing symptom deterioration with a combined “clinical psychiatrist criteria” and BPRS rating deterioration in core positive symptoms criteria (Linszen et al., 1996), and one study (Miklowitz et al. (2014) reporting on the number of people who converted to psychosis as assessed by a specified deterioration on the positive symptom scale on the Scale of Prodromal Symptoms (SOPS; Hawkins et al., 2004). FIp was more likely to reduce the risk of relapse than comparison groups by the end of treatment, and this effect was of a medium size (*k* = 7, *RR* = 0.58, 95 % CI [0.34, 1.00]). However, relapse prevention gains were not maintained at follow up. Closer inspection of the studies included in this follow-up meta-analysis revealed that only Chien et al. (2016a) showed sustained gains in terms of risk of relapse at a 6 month follow, whereas Leavey et al. (2004) and Rossberg et al. (2010) with 9-month and 5-year follow ups respectively failed to show sustained gains. (Please see Table 3b in Supplementary Materials). There was no evidence that family intervention achieved reductions in *hospital admission length*.

#### Hypothesised mechanisms of change in family intervention

Family intervention was more likely to reduce the number of carers with *high expressed emotion* ratings by the end of treatment (as measured by the Camberwell Family Interview; Leff and Vaughn, 1985 clinician ratings and the Family Questionnaire; Wiedemann et al., 2002) (*k* = 2, *OR* = 16.76, 95 % CI [1.96, 143.44]), as well as diminishing the amount of expressed criticism by the end of treatment, with a large effect size (*k* = 3, *d* = −0.84, 95 % CI [−1.15, −0.53]), with one study (Gleeson et al., 2010), suggesting gains could be maintained up to a 2.5 year follow up (*d* = −0.96, 95 % CI [−1.87, −0.05]). Similarly, *conflict communication* was more likely to be reduced in those attending FIp in comparison to control groups, with a medium effect size (*k* = 3, *d* = −0.44, 95 % CI [−0.77, −0.12]). Family intervention did not affect ratings of carer emotional over-involvement.

#### Carer outcomes

*Carer burden*, assessed by the Experience of Caregiving Inventory (ECI, Szmukler et al., 1996), was reduced and *carer well-being* (as assessed by the GHQ-28, Goldberg, 1978 and K-10, Kessler and Mroczek, 1992) improved by the end of family intervention with large effect sizes (burden: *k* = 3, *d* = −0.72, 95 % CI [−0.97, −0.47]; well-being: *k* = 2, *d* = −1.09, 95 % CI [−2.07, −0.12]) but these improvements were not observed at follow up.

#### Heterogeneity and sensitivity analyses

Nine of the 15 meta-analyses had high levels of heterogeneity as assessed by an I^2^ higher than 75% and/or a significant χ2 test (See last two columns in Table 3). Of those, five included at least *k* = 3 studies and therefore the one-study-removed method was applied to ascertain if statistical heterogeneity could be reduced to acceptable levels. The impact of removing one study on each of the meta-analyses is reported below alongside an evaluation of potential clinical or methodological heterogeneity that would justify accepting removal of that particular study in relation to other studies remaining in the meta-analyses. Please see Supplementary Materials for further details.

##### Service user outcomes

For the general functioning at the end of treatment outcome, the removal of Zhang et al. (1994) reduced heterogeneity substantially (I^2^ = 9%). The effect size and 95% CI were 0.48 [0.91, 0.92] (*k* = 3), favoring FIp, as was the case when Zhang et al. (1994) was included. This study only examined male service users which could have contributed to heterogeneity and limited generalizability of their findings to females. Moreover, Zhang et al. (1994) looked at first admission (mean age 24) whereas two of the remaining two studies (Rund et al., 1994; Calvo et al., 2015) focused on very early onset samples (under 18's). Although the targeted populations differed, there seems to be insufficient grounds for its removal, given that Zhang et al. (1994) quality, as assessed by the EPHPP, was not particularly weak. In relation to the relapse at follow up outcome, the removal of Chien et al. (2016a) eliminated heterogeneity (I2 = 0%). The effect size and 95% CI were 1.50 [1.02, 2.22] (*k* = 2), favoring FIp, which was not the case when this study was included. However, given that Chien et al. (2016a) had a strong quality rating and that these authors delivered single-family groups, as another of the studies included in this meta-analysis (Leavey et al., 2004), the decision was not to exclude it from the analysis.

##### Mechanisms outcomes

The removal of McCann et al. (2013) reduced heterogeneity in the meta-analysis for *EE criticism at the end of treatment* (I^2^ = 30%). The effect size and 95% CI were 0.07 [−0.38, 0.52] (*k* = 2), no longer favoring FIp. However, McCann et al. (2013) had the strongest quality rating amongst the three studies and therefore it was decided not to remove it.

##### Carer outcomes

It was not possible to reduce heterogeneity by removing any of the three studies in the *carer burden at end of treatment* meta-analysis but for *follow up* outcome, removal of Chien et al. (2016b) reduced heterogeneity (I^2^ = 0%). The effect size and 95% CI were 0.31 [−0.03, 0.65] (*k* = 2), favoring FIp, which was not the case, with its inclusion. As above, a final decision to keep Chien et al. (2016b) in the latter meta-analysis was made given that this study had a strong quality rating, and was a replication of McCann et al. (2013) in China.

In sum, although high heterogeneity was identified in a range of meta-analyses, evaluation of clinical and methodological characteristics resulted in the decision to not remove any of the included studies.

## Discussion

This systematic review and meta-analysis aimed to answer key questions about the efficacy of family interventions in early psychosis and their outcomes for service users and relatives. All studies examined FIp as an adjunct to standard care and/or pharmacological treatment in comparison to a control group (primarily, standard care alone). The review yielded 17 papers from 14 distinct trials. The findings suggested FIp may have an important role in reducing the number of relapses and increasing general functioning at the end of treatment for those with early psychosis. There was evidence for change in the quality of the family environment with carers more likely to shift from high to low EE ratings at the end of intervention and less likely to engage in conflict-laden communication styles. There was also evidence that FIp improved carers' positive well-being and reduced burden of care in comparison to standard care although these effects were not sustained at follow up.

Overall, there was no significant reduction in symptoms or in the number of hospital admissions between FIp and comparison groups at the end of treatment although a delayed effect in symptom reduction was found at follow up.

### Is family intervention effective in reducing relapse in early intervention?

There was promising evidence for significant reductions in relapse rates, a finding that replicates the literature examining FIp in mixed-duration schizophrenia-spectrum disorders (e.g., Pharoah et al., 2010) and a previous review examining early psychosis (Bird et al., 2010). FIp's relapse reduction effectiveness with the early psychosis group is encouraging, particularly given the elevated relapse rates in early psychosis populations and their links with poorer long term outcomes, which include personal and familial distress (Ho et al., 2003) and disrupted social and vocational development in young people (Penn et al., 2005). Preventing or reducing relapse is therefore an important goal of FIp. The current findings, however, indicate that reductions in relapse risk were not sustained at follow up. Although one of the three studies included in this meta-analysis showed sustained gains at 6 months, the remaining two (Leavey et al., 2004; Rossberg et al., 2010), with 9-month and 5-year follow-ups, indicated improvements were not sustained in the long run. Further research is needed to ascertain the reasons, particularly in the context of recent research highlighting sustained outcome follow up in longer term psychosis groups (Ran et al., 2015). The current findings may speak to the importance of providing top up (booster) sessions to support service users and families in managing the impact of psychosis and addressing the factors that may increase the service user's vulnerability to relapse.

This review highlighted no significant differences in symptom reduction at the end of FIp treatment. This is broadly similar to findings from the mixed-duration schizophrenia spectrum disorders, where results for symptom change were generally equivocal although did favor FIp on some outcome measures (Pharoah et al., 2010). It is of note that this review only analyzed overall symptom reduction and it may be useful to examine positive and negative symptoms separately particularly given previous findings that link FIp to improvements in social functioning (Pharoah et al., 2010). General functioning was found to be improved in FIp groups when compared to controls. One study noted that service users with the lower levels of psychosocial functioning at the start of treatment yielded the largest benefits from FIp (Rund et al., 1994). Improvements in social functioning have traditionally been linked to the common practice in FIp of supporting the service user and their relatives to modify specific behaviors which, for many families, may lead to service users engaging in more activities including social and vocational opportunities. However, further research is required to identify the mechanisms for change (e.g., Giron et al., 2010).

### Improvements in targeted mechanisms: criticism in high expressed emotion

This review showed that FIp reduces high EE within an early psychosis sample. There was evidence of reduced expression of criticism/hostility but no evidence of a change in reports of high emotional over-involvement. Findings in the broader schizophrenia-spectrum literature also suggested that FIp improves levels of EE (Pharoah et al., 2010), however this included emotional over-involvement. Criticism from carers has been positively associated with increases in levels of anxiety in service users (Kuipers et al., 2006; Docherty et al., 2011). The reductions in criticism remains important given its predictive role in subsequent relapse (Bebbington and Kuipers, 1994) and adverse impact on patient well-being including at a neural level (Rylands et al., 2011). The failure to identify significant reductions in levels of EOI may, in part, reflect the complexity of the concept (and measurement) of EOI and difficulties identified within the literature of distinguishing from behaviors and attitudes that can be understood as being caring (van Os et al., 2001; Singh et al., 2013). Early psychosis is characterized by high levels of carer and patient distress and fluctuating symptoms, which may contribute to EE being particularly unstable, changing over time or in relation to stressors rather than intervention (Patterson et al., 2005). Symptom severity or duration were not typically controlled for across the studies, thus limiting the conclusions that can be made in this regard.

Future work is needed to understand the mechanisms of each component of EE (particularly high EOI) in order to prevent the entrenchment of high EE behaviors and responses in the long-term. It is likely that until we understand these mechanisms, current interventions for the early psychosis group may be limited in their effectiveness. One aspect of the family environment that was not measured in the current studies, but may benefit from future research was warmth. If FIp improved carers' experiences, this may impact on aspects of the family environment not captured by current measures (for example an increase in positive caregiving appraisals and readiness to continue providing care (e.g., Berglund et al., 2003). Furthermore, one study indicated that caution should be exercised in offering FIp (specifically communication training and problem-solving) to low EE families (Linszen et al., 1996). The authors highlight that offering interventions such as communication training when this is not a problematic area for a family may be perceived as invalidating and critical, thus increasing stress and adversely affecting relapse (Linszen et al., 1996). It is important to recognize that not all families will require intervention or find it acceptable (Cohen et al., 2010; Onwumere et al., 2016). Bhugra and McKenzie (2003) reviewed the cross cultural literature on EE and noted some families view FIp as somewhat intrusive and prescriptive, whereas others find it a useful way to learn more about supporting their relative through the illness. It may be important to fully assess carers' needs and wishes before offering FIp.

### Carer burden and well-being: need for ongoing support?

This review found evidence for improvements in carer psychological health and/or general well-being. It is known that carers of individuals with early psychosis experience high levels of distress and related health problems (Addington et al., 2003; Boydell et al., 2014; Jansen et al., 2015; Onwumere et al., 2015), which can persist (Lee et al., 2006). In their qualitative investigation of 80 early psychosis carers, Lavis et al. (2015) noted that carers of people with early psychosis describe an ongoing level of distress and a continual adjustment process. They suggest that the distress can remain long after the service user recovers, as carers' lives have often been greatly impacted by the first experience of psychosis in the family. Lavis et al. (2015) also note that carers often reported they were not asked by the service about how they themselves were managing and feeling. It is possible that FIp is effective in providing information about psychosis and practical issues whilst also addressing carers' own levels of distress and the emotional experience of caregiving to some degree.

There was also evidence that FIp improves the appraisal of caregiving, with studies observing reductions in negative caregiving experiences (burden). These findings replicate other studies examining the wider schizophrenia spectrum (Giron et al., 2010). It could be argued that FIp allows families to feel more supported in their caregiving experiences which in turn impacts on their subjective appraisals of caregiving. Qualitative research suggests that carers of people with early psychosis find components of FIp such as information around psychosis and medication management important in helping to increase their confidence in supporting their relative (Nilsen et al., 2014; Lavis et al., 2015).

In order to sustain improvements in caregiving burden and carer well-being, a range of alternative interventions could be investigated. For example, peer-led mutual support groups for carers might be of particular interest (Chien and Norman, 2009; Chien and Chan, 2013).

### Limitations

A small number of studies (*k* = 2–4) were included in each of the meta-analysis. There was also high heterogeneity amongst included studies. There were differences with regard to patient characteristics (including age of symptom onset, duration of untreated illness and baseline symptom severity) alongside differences in the characteristics and components of the interventions (which varied in content, structure and duration). This restricts the conclusions that can be drawn about the specific components of FIp that might be most effective for whom. Furthermore, the nature of comparison groups was highly variable. Several trials examined in this review described specialist early intervention for psychosis services as standard care (Gleeson et al., 2010; Rossberg et al., 2010; McCann et al., 2013; Calvo et al., 2014; O'Brien et al., 2014 and Miklowitz et al., 2014). These generally comprised set treatment protocols including optimal pharmacotherapy and a range of psychoeducational and psychosocial interventions, often including individual psychotherapy if required. This may mean that some effects of FIp are concealed. For example, one study did not find differences between groups, but noted both FIp and the standard care control groups demonstrated lower relapse rates in comparison to those found in the wider literature (Linszen et al., 1996). They suggest that the highly specialist nature of these services is likely to have been an effective intervention in its own right, thus making it difficult to demonstrate any further benefit of FIp (Linszen et al., 1996). It may be that shared components of FIp and specialist care (such as regular contact with a team) allow a family to feel supported more generally. Alternatively, there may be similar outcomes but different mechanisms for change. For example, FIp may improve symptoms via warmth and problem-solving, whereas specialist services improve symptoms via medication management and/or contact with care coordinators.

In addition, due to the ethics related to withholding effective treatment, some studies offered a limited number of family psychoeducational sessions in control conditions, which again might mean the full impact of FIp is underestimated in these studies. Conversely, it was not possible for the comparison groups to control for non-specific factors such as the number of face-to-face contacts or being in a group. For example, there is evidence that support groups have been shown to be particularly beneficial for carers of people with early psychosis (Chien and Norman, 2009). Further, research is needed to determine the active ingredients of FIp within the early illness phases.

### Engagement with family intervention

Poor intervention uptake, moderate intervention completion and high study dropout rates were a feature of some trials, particularly those that included longer follow-up periods. There are likely to be significant differences between those who engage in treatment and follow-up and those who drop out, thus potentially biasing the results in the included trials. For example, Nugter et al. (1997) noted that the families who completed FIp were generally a well-functioning group who had engaged throughout, which may have meant there was little room for further improvement to be captured. There is a limited understanding of the variables that may influence the engagement of carers with Early Intervention for Psychosis services. It is important to understand the barriers to engagement and identify the specific needs of early psychosis families to determine the factors that may help promote better engagement with services when they are required (Nilsen et al., 2015).

### Clinical implications and future research

Given the evidence synthesized in this review, we support recommendations for the provision of FIp in early intervention. However, we cannot make specific recommendations regarding the optimal components of FIp for early psychosis, given the heterogeneity of included trials and the specific focus of this review on carer and patient outcomes (rather than the intervention components). Further, research is required to identify what FIp components and in what combination yield specific positive outcomes.

Seeking feedback from carers might also help to shape interventions to meet carers' unmet needs. Leavey et al. (2004) note that at the very early stages of a psychosis, caregivers requested more practical support, such as details on welfare benefits or how to access services. It has been suggested that carers know “how much” they need, rather than interventions being prescribed for them (Leavey et al., 2004) and that families might adjust their involvement with services and interventions in line with the intensity of symptoms (Gleeson et al., 2010). Research on the efficacy of needs led intervention during the FEP phase is indicated (Sellwood et al., 2007; Roddy et al., 2015). It may be important to develop services for carers that are more carer-informed and carer-led, rather than assuming generic protocols (Sin et al., 2007; Kuipers et al., 2010). It may be helpful for early intervention services to adopt a triage system to assess relatives' needs and to have a range of flexible interventions available, including low-intensity approaches such as information leaflets alongside more intensive and therapeutic family support options (Cohen et al., 2010; Onwumere et al., 2016). Internet-based interventions such as bibliotherapy also deserve further attention, given reported high retention rates (McCann et al., 2013 and Chien et al., 2016a,b).

Crucially, few of the included studies utilized Black and Minority Ethnic samples, who are over represented in rates of psychosis in many regions (Morgan et al., 2010). Further research therefore needs to specifically investigate the acceptability and efficacy of Family Intervention in this population (Edge et al., 2016), particularly in the context of migration and higher social adversity.

## Conclusions

FIp generally aims to increase familial understanding of relapse indicators, helping relatives to engage in supportive patterns of responding, thus preventing relapse and hospitalization (Onwumere et al., 2011). This review showed evidence of improvement in carer outcomes, and indications of reduced relapse rates, although this finding was not sustained at follow-up, particularly those beyond 6 months. It is possible that FIp helps carers support their family members more by providing information and guidance on the practical tasks and assisting with treatment engagement. Evidence suggests that carers' own needs and the emotional impact of caregiving are a neglected area and FIp showed improvements in carers' well-being and a reduced sense of burden. Further, understanding of carer adaptation to the first onset of psychosis in a loved one is required alongside ascertaining the type of intervention and active ingredients that are most effective for whom. Such research can then inform the development of theoretically driven yet tailored interventions that meet the specific needs of carers of early psychosis.

## Author contributions

MC conducted the research project as part of a Doctorate in Clinical Psychology. MFA and JO were her supervisors. All authors were involved in the conceptualization and design of the project. MC was in charge of project administration and data collection. MC and MFA conducted the analyses. All authors were involved in writing the manuscript.

### Conflict of interest statement

The authors declare that the research was conducted in the absence of any commercial or financial relationships that could be construed as a potential conflict of interest.
