# Supplementary material for: Do Family Interventions Improve Outcomes in Early Psychosis? A Systematic Review and Meta-Analysis
Source: Front Psychol. 2017 Mar 27;8:371. doi: 10.3389/fpsyg.2017.00371 (PMC5366348; doi:10.3389/fpsyg.2017.00371)
Supplement: Supplementary file 1 [file DataSheet1.DOCX]

Supplementary Material

Article Title: Do Family Interventions Improve Outcomes in Early Psychosis? A Systematic Review and Meta-Analysis

Authors: Melanie Claxton^1^, Juliana Onwumere^2^, Miriam Fornells-Ambrojo^1*^

*** Correspondence:**: Miriam Fornells-Ambrojo; Research Dept of Clinical, Educational and Health Psychology University College London; 1-19 Torrington Place; London WC1E 7HB, United Kindgdom; e-mail: miriam.fornells-ambrojo@ucl.ac.uk

# Supplementary Data: Main meta-analyses

Supplementary materials: Details of individual meta-analyses

**Client outcomes:**

1) Client symptoms

1a) Symptom reduction at end of treatment


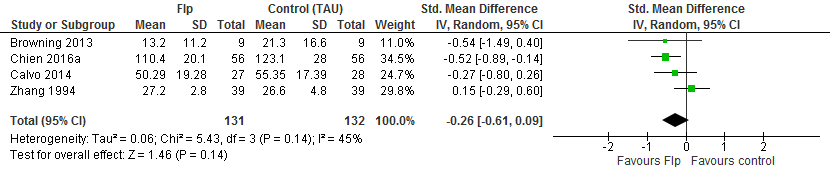


1b) Symptom reduction at follow up:


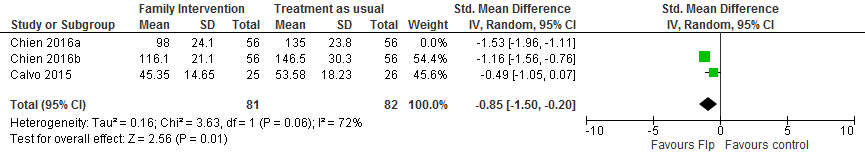


2) Client functioning:

2a) Functioning at end of treatment:


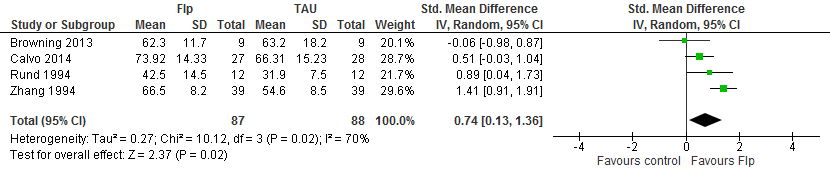
2b) Functioning at follow up:


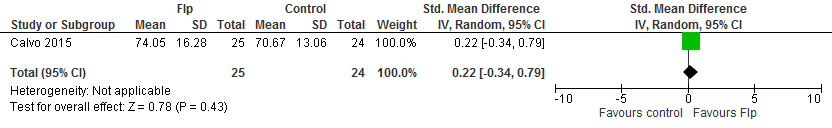


3) Relapse (number of people hospitalised, relapse as assessed by symptom deterioration, transition to psychosis)

3a) Relapse (number of people hospitalised, relapse or transition to psychosis, transition to psychosis)) by the end of treatment


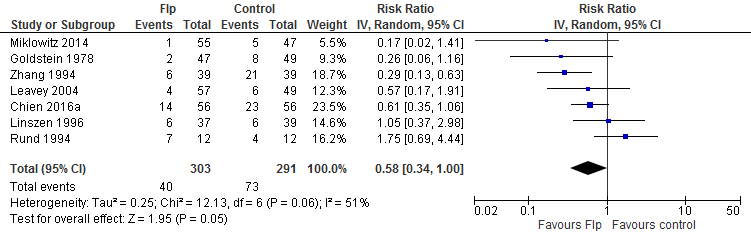


3b) Relapse (number of people hospitalised) by follow up


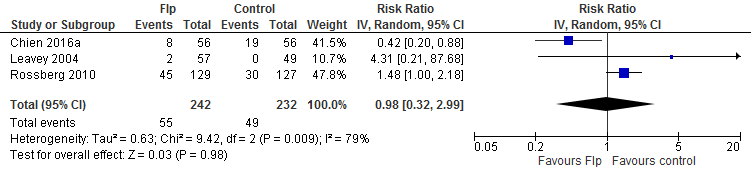


4) Number of days in hospital

4a) Number of days in hospital by the end of treatment


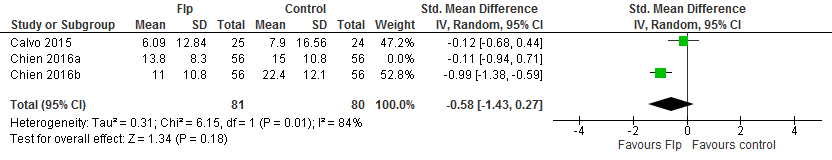


4b) Number of days in hospital by the follow up


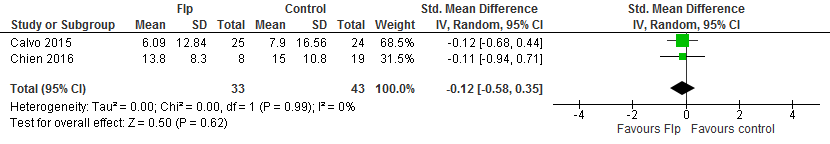


Carer outcomes

5) Expressed emotion

5a) Number of carers who changed from high to low EE by the end of treatment


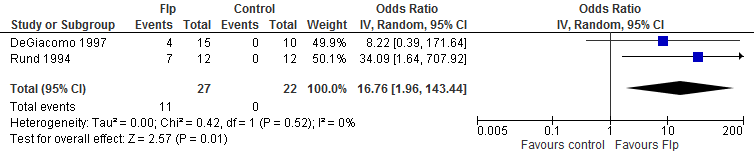


6A) Expressed emotion: Mean number of critical/ hostile comments at the end of treatment


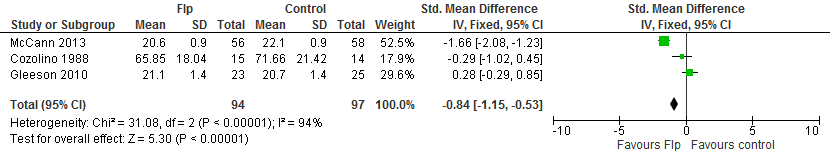


6B) Expressed emotion: Mean number of critical/ hostile comments at follow up


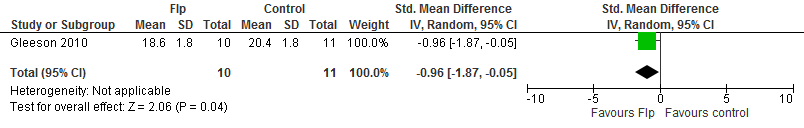


7a) Expressed emotion: Emotional over-involvement by the end of treatment
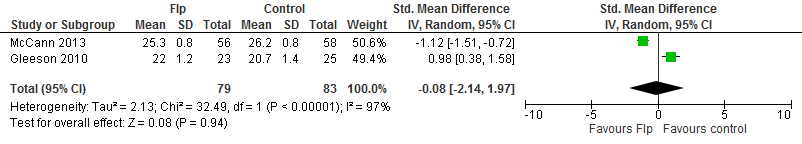


7b) Expressed emotion: Emotional over-involvement by follow up
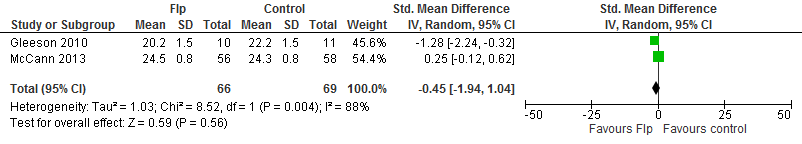


8 ) Communication conflict:
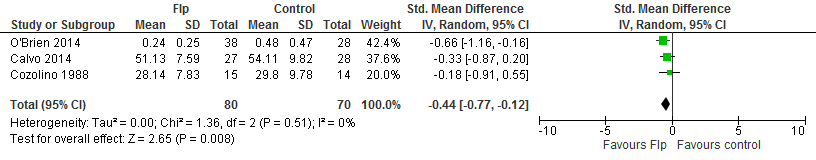


9) Caregiver burden

9a) Caregiver burden by the end of treatment


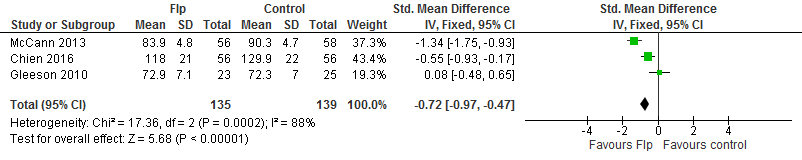


9b) Caregiver burden at follow up


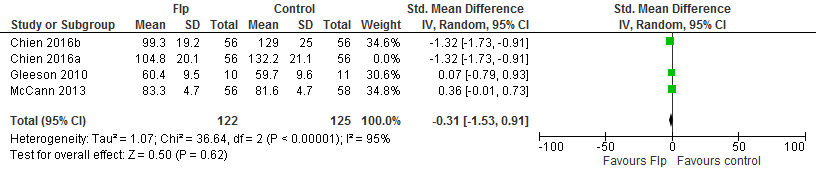


10) Caregiver wellbeing

10a) Caregiver wellbeing by the end of treatment


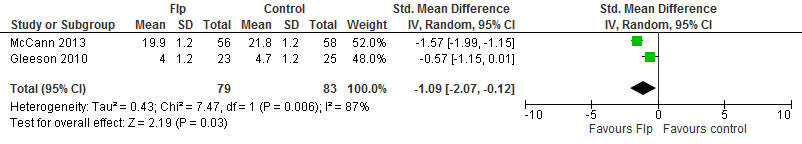


10b) Caregiver wellbeing at follow up


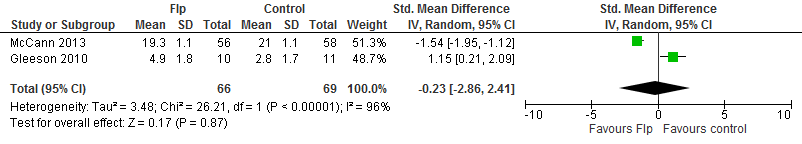


# Supplementary Data: Sensitivity analyses

Supplementary materials: Sensitivity analyses

Details of individual meta-analyses

**Client outcomes:**

2a) Functioning at end of treatment:

The only study whose removal resulted in reduction in heterogeneity was Zhang (1994)


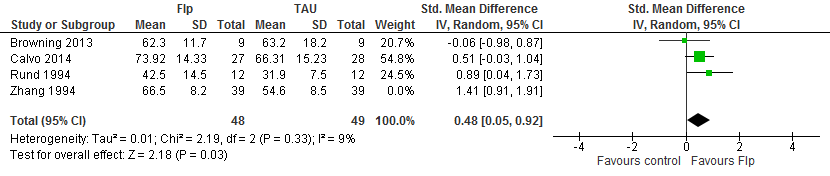


3b) Relapse (number of people hospitalised) by follow up

Chien (2016a)’ removal significantly reduced heterogeneity to 0%. After the removal of this study, the risk of relapse was more likely to decrease in the FIp group compared to control.


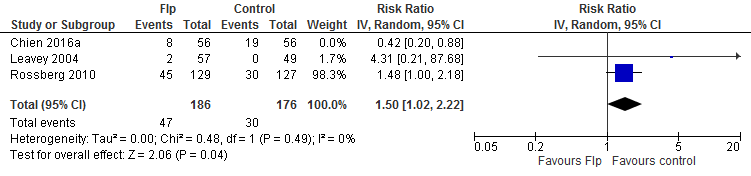


Carer outcomes

6a) Expressed emotion: Mean number of critical/ hostile comments at the end of treatment

MacCann (2013) significantly reduces heterogeneity, when this study is removed, FIp is found to have no superior effect on reduction of critical comment by the end of treatment.


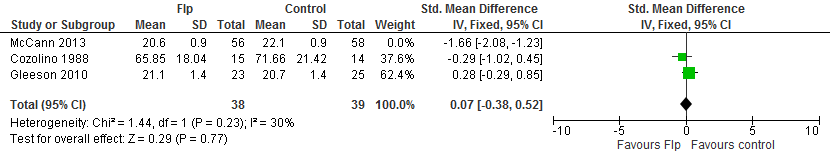


9a) Caregiver burden by the end of treatment

No study was found to contribute to heterogeneity, removal of each of them still resulted in I^2^score>70%

9b) Caregiver burden at follow up

Removal of Chien (2016b) resulted in a reduction of heterogeneity. When this study is removed, FIp is found to sustain reductions in caregiver burden at follow up.


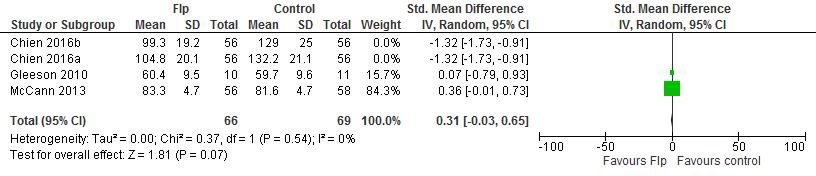


# 
